# The introduction of new hosts with human trade shapes the extant distribution of *Toxoplasma gondii* lineages

**DOI:** 10.1371/journal.pntd.0007435

**Published:** 2019-07-11

**Authors:** Lokman Galal, Amedine Sarr, Thomas Cuny, Carine Brouat, Fatoumata Coulibaly, Mbacké Sembène, Moustapha Diagne, Mamoudou Diallo, Aliou Sow, Azra Hamidović, Nicolas Plault, Marie-Laure Dardé, Daniel Ajzenberg, Aurélien Mercier

**Affiliations:** 1 INSERM, Univ. Limoges, CHU Limoges, UMR 1094, Tropical Neuroepidemiology, Institute of Epidemiology and Tropical Neurology, GEIST, Limoges, France; 2 CBGP, IRD, CIRAD, INRA, Montpellier SupAgro, Univ. Montpellier, Cedex, France; 3 Laboratory of Parasitology-Mycology, Faculty of Medicine and Pharmacy, University of Cheikh Anta Diop, Dakar, Senegal; 4 Département de Biologie, Unité de formation et de recherche en Sciences Biologiques, Université Péléforo Gon Coulibaly, Korhogo, Côte d'Ivoire; 5 Département de Biologie Animale, Faculté des Sciences et Techniques, Université Cheikh Anta Diop (UCAD), Dakar, Senegal; 6 BIOPASS, CBGP-IRD, ISRA, UCAD, Dakar, CP, Senegal; 7 Centre National de Référence Toxoplasmose/*Toxoplasma* Biological Resource Center, CHU Limoges, Limoges, France; Imperial College London, UNITED KINGDOM

## Abstract

*Toxoplasma gondii* is a zoonotic protozoan with a worldwide occurrence, but the determinants of the current pattern in the geographical distribution of *T*. *gondii* lineages and strains remain poorly understood. To test the influence of human trade on *T*. *gondii* populations, we conducted a population genetic study of 72 *T*. *gondii* animal isolates from Senegal, a West African country in which the ongoing inland progress of invasive murine hosts (introduced in port cities of Senegal since the 16^th^ century by European sailors) is well described. Isolates were mainly collected on free-range poultry, which are considered as relevant bioindicators of *T*. *gondii* strain diversity in the domestic environment. Sampling was conducted in two port cities of Senegal (Dakar and Saint-Louis) and in one inland region (Kedougou). Population genetic analyses using 15 microsatellite markers revealed different patterns between port cities where lineages non-virulent for mice (type II, type III, and Africa 4) were predominant, and Kedougou where the mouse-virulent Africa 1 lineage was the most common. By considering the current spatial pattern in the inland progress of invasive rodents in Senegal, our results suggest that the invasive house mouse *Mus musculus domesticus* counter-selects the Africa 1 lineage in the invaded areas. The comparison of the microsatellite alleles of type II strains from Senegal to type II strains from other areas in Africa and Western Europe, using discriminant analysis of principal components and Network analysis, point to a mainly Western European origin of the type II lineage in Senegal. Collectively, these findings suggest that human-mediated intercontinental migrations of murine hosts are important vectors of *T*. *gondii* strains. Differential susceptibility of endemic and introduced murine hosts to various *T*. *gondii* strains probably determines the persistence of these strains in the environment, and therefore their availability for human and animal infection.

## Author summary

*Toxoplasma gondii* is a zoonotic protozoan with a worldwide distribution and which can infects virtually all warm-blooded species, including human. Clinical expression of human toxoplasmosis, as well as *T*. *gondii* strains diversity, exhibit contrasting patterns across geographic regions. The determinants of this geographical structure are poorly understood, but a growing body of evidence supports an important role of human-mediated migrations of *T*. *gondii* hosts in the intercontinental dissemination of some parasite lineages. The results of our study conducted in Senegal suggest that the invasive house mouse—which was introduced in the port cities of this country through maritime trade since colonial times—has a dramatic influence on the *T*. *gondii* populations of invaded areas. This important *T*. *gondii* reservoir seems to be a vector for the intercontinental migrations of *T*. *gondii*. In addition, it may have a role in the selection (or the counter-selection) of local *T*. *gondii* populations found in invaded areas. This study provides insights into the mechanisms shaping *T*. *gondii* populations, thereby determining which strains will be available for human and animal infection.

## Introduction

*Toxoplasma gondii* is a zoonotic protozoan with a worldwide distribution. Felids are the only final hosts and all other species of mammals and birds are intermediate hosts. Within the domestic cycle, infection can occur through the ingestion of few of the million oocysts shed in the environment by cats during the three to 14 days following their primary infection [1]. Infected hosts often develop persistent cysts in their tissue, which constitute the main source of infection for cats and an important potential source of infection for meat-consuming intermediate hosts, including humans. The genetic diversity of *T*. *gondii* strains shows a strong geographical structure [2–4]. This geographical pattern is of epidemiological importance because *T*. *gondii* genotype has often been associated with disease severity in immunocompetent individuals, especially in South America [5–10]. Few data are available for Africa, but some indirectly suggest a significant burden of ocular toxoplasmosis in West and Central Africa [11,12]. Evidence of an important role of human-mediated dispersal in shaping *T*. *gondii* population structure is supported by the intercontinental occurrence of some lineages [13]. Mainly, the remarkable success of the archetypal type II and type III lineages in global spread has been attributed to human exchanges through movements of infected livestock and involuntary dispersal of infected rodents via maritime or terrestrial routes [14,15]. The intensification of maritime trade since the sixteenth century has probably given strains of type II and type III lineages the opportunity to spread from Western Europe to new lands such as America and Australia, but also to West and Central Africa. [4,14,16]. Based on these assumptions, Lehmann et al. [14] speculated that the genetic background of *T*. *gondii* strains near ports that were active during early transatlantic trade should differ markedly from that in regions distant from such ports.

The aim of the present study was to test this hypothesis in Senegal (West Africa). Unlike North and East Africa where the intercontinental lineage type II, followed by type III, are by far the predominant lineages, West and Central Africa seem to be the refuge for a more autochthonous diversity of African *T*. *gondii* strains, mainly composed of strains of the Africa 1 lineage [4]. This pattern has been attributed to the more recent exposure of West and Central Africa to the influence of globalization compared to North and East Africa that have anciently been linked to Europe and Asia through privileged trade exchanges during the successive historical periods. West Africa appears therefore to be a suitable framework to test the influence of relatively recent human exchanges on *T*. *gondii* population structure. In this study, we compared the diversity of *T*. *gondii* strains circulating among domestic animals in the port cities of Saint-Louis and Dakar, with those circulating in the Kedougou region inland, which is located more than 500 kilometres from the coast. Most samples were collected on domestic poultry (mainly chickens). These intermediate hosts live in the vicinity of human dwellings and are considered as good sentinels for *T*. *gondii* occurrence in the environment given that they feed on the ground and that they rarely become sick from *T*. *gondii* infection [17–19].

In order to evaluate the extent of gene flow between *T*. *gondii* populations from Senegal and other regions of the world through both terrestrial and maritime routes, we compared type II *T*. *gondii* strains from Senegal to those from other areas in Western Europe and Africa.

## Materials and methods

### Study area and *T*. *gondii* strains isolation

From April 2016 to April 2018, three regions of Senegal were investigated for *T*. *gondii* strain isolation: the coastal regions of Dakar and Saint-Louis, which were founded during the colonial period by French sailors, and the inland region of Kedougou. In each of these regions, sampling was conducted in both urban and rural localities. Our sampling efforts focused mainly on backyard poultry raised around households and were occasionally completed by opportunist sampling of other domestic or wild animals when they were available. The geographic origin of each animal included in this study was checked by questioning the owners to insure that infection had occurred locally. All sampled households were georeferenced using a Juno SC GPS Data Collection PDA (Trimble, California, USA). Blood samples were collected from poultry from the wing vein for serological screening. For animals sampled opportunistically, as in the case of home slaughter or for animals found freshly dead by the roadside, blood samples were collected during slaughtering or from blood clot in the heart during post-mortem examination. After separation by centrifugation, sera were tested for presence of antibodies against *T*. *gondii* using the modified agglutination test (MAT) with a seropositivity cut-off at 1:20 dilution titer [20]. Overall, 2,040 animals were sampled, the majority being chickens (79.5%) and ducks (14.8%) (S1 Table). The total seroprevalence was 11.8% (241/2040; 95% confidence interval CI: 10.4%—13.2%). According to poultry’s availability for sale, a total of 122 seropositive domestic birds were purchased, brought alive to the Institut de Recherche pour le Développement (Belair, Dakar) and euthanized. Brain and heart samples were collected and kept at 4°C before being processed for parasite isolation. In addition, brain and/or heart samples of 33 others seropositive animals (S1 Table) were also kept at 4°C before processing.

The isolation protocol was performed as reported previously [21]. Brain and heart samples of each animal were homogenized together using a blender in saline solution (0.9% NaCl) containing 0.4% of trypsin and 40μg/ml gentamycin and incubated in a shaker water bath at 37°C for 90 min. The suspensions were filtered through two layers of gauze and washed three times by centrifugation for 10 min at 2600 rpm. The obtained digestates were then re-suspended in saline solution and treated with an antibiotic saline solution (1000 U/ml penicillin and 100 μg streptomycin/ml in saline solution). The digestates were intraperitoneally inoculated into three out-bred female Swiss Webster (SW) mice (1 mL/mice) provided by the Institut Pasteur of Dakar. All inoculated mice were monitored daily for clinical signs of toxoplasmosis during four weeks. Ill mice developing ascites were punctured for peritoneal exudates to check for the presence of tachyzoites before being euthanized. After four weeks, surviving mice were tested for *T*. *gondii* antibodies by MAT serology (cut-off at 1:20 serum dilution). Seropositive mice were euthanized and brain samples were homogenized with 1 ml of physiological solution for microscopic examination of tissue cysts. For each sample, 200 μl of peritoneal exudate or brain homogenate was stored at -20°C for DNA extraction. Live parasites were cryopreserved in liquid nitrogen with RPMI containing 10% FCS and 10% DMSO and were sent to the *T*. *gondii* Biological Resource Centre (BRC), Limoges, France, (http://www.toxocrb.com) for strain preservation. The isolation protocol was approved and accepted by the Research Ethics Committee of Cheikh Anta Diop University in Senegal (Registration numbers: 0232/2017/CAR/UCAD and 0278/2018/CAR/UCAD).

Cryopreserved brain samples for which no tissue cysts could be observed were re-inoculated into SW mice in Limoges, France. After 4 weeks, mice that tested seropositive using MAT serology were euthanized. Their brains were aseptically sampled, rinsed in saline solution, placed in 1 ml of saline solution, and extruded through a 21-gauge needle several times, and then through a 23-gauge needle. Half of this suspension was treated by 1 ml of trypsin-EDTA solution (pre-heated at 37°C), thoroughly shaken, and incubated at 37°C for 3 minutes to disrupt tissue-cysts. The obtained suspension was then re-extruded through a 25-gauge needle several times, washed in 5 ml of Iscove's Modified Dulbecco's Medium (IMDM), resuspended in 1ml of IMDM, and inoculated in a Vero cell monolayer in a T75-flask. The culture medium was composed of IMDM treated with 1% of antibiotic saline solution (1000 U/ml penicillin and 100 μg streptomycin/ml in saline solution) and enriched with 2% of fetal bovine serum (FBS). Parasite growth was observed between one and four weeks post-initial inoculation. Animal experimentation conducted in Limoges was approved and accepted by the Ethics Committee for Animal Experimentation n°033 validated by the French Ministry of National Education, Higher Education and Research (Registration numbers: APAFIS#14582-2018041010294175 v2).

All experimental procedures were conducted according to European guidelines for animal care (‘‘Journal Officiel des Communautés Européennes”, L358, December 18, 1986).

### DNA extraction and microsatellite genotyping

Total genomic DNA was extracted from 200μl of mice brain homogenates, mice ascites or supernatants of cell culture, using the QIAamp DNA MiniKit (Qiagen, Courtaboeuf, France). For animal samples that did not infect laboratory mice, DNA extraction was performed directly on 200μl of animal tissue digestate. *Toxoplasma gondii* strains were genotyped using 15 microsatellite markers distributed on 11 of the 14 chromosomes composing *T*. *gondii* genome in a single multiplex PCR-assay, as described previously [22]. Those 15 loci included a combination of eight “typing” markers with low polymorphism (*TUB2*, *W35*, *TgM-A*, *B17*, *B18*, *M33*, *IV*.*1* and *XI*.*1*) that show little or no variation within lineages and seven “fingerprinting” markers with high polymorphism (*M48*, *M102*, *N83*, *N82*, *AA*, *N61*, *N60*) that show significant variation within lineages [23]. For each strain successfully genotyped at some loci but not at others, each failed locus was amplified separately by simplex PCR (to prevent primer competition) using the same protocol as the multiplex PCR-assay. PCR products were sized using capillary electrophoresis on ABI PRISM 3130xl (Applied Biosystems, Foster City, CA) and the GenScan 500 ROX dye size standard (Applied Biosystems). Results were analyzed using GeneMapper 5.0 software packages (Applied Biosystems).

### Assignment of Senegalese *T*. *gondii* strains to clonal lineages

To assign each strain to a clonal lineage, Senegalese multilocus genotypes (MLGs) were compared to those from reference strains representative of the main *T*. *gondii* clonal lineages previously described worldwide. Those reference strains are single nucleotide polymorphism (SNP) inferred lineages from previous studies, either based on multilocus sequence typing (MLST), whole genome sequencing (WGS), or multilocus restriction fragment length polymorphism (RFLP) analysis (S2 Table). Assignment to a clonal lineage relied on the examination of the allelic combination at eight “typing” alleles that constitutes the lineage identity for each strain [22]. In order to further confirm the relationships of Senegalese MLGs with the reference *T*. *gondii* lineages, an unweighted pair group method with arithmetic mean (UPGMA) dendrogram was generated by including all MLGs from Senegal with a single reference MLG for each of the major clonal lineages that were identified worldwide (S2 Table). This UPGMA dendrogram was produced using the BRUVO.BOOT function (based on Bruvo’s genetic distance) with 1,000 bootstrap replications, implemented in the “Poppr” package [24] in R version 3.4.0. This package is specifically designed for analysis of clonal, sexual or admixed populations, that may not fit to basic assumptions of the Wright–Fisher model of populations, which implies panmixia and Hardy–Weinberg equilibrium.

The software QGIS V2.14.14-Essen [25] was used to map the geographical distribution of the sampling locations and the corresponding genotypes.

### Geographical structure

To estimate the occurrence of a geographical structure within *T*. *gondii* populations, an AMOVA was performed using GenAlEx 6.51 software package [26]. The individuals were grouped according to their geographical origin. The genetic differentiation between geographical populations was determined using a pairwise population test (*PHI*_PT_). *PHI*_PT_, an analogue of the fixation index *F*_ST_, suppresses the within-population variance and ranges from 0 (no differentiation) to 1 (full differentiation). Levels of significance were determined by computing 10,000 random permutations.

### Genotyping and genetic diversity

Genotypic diversity indices (Stoddart and Taylor’s index; Simpson’s index; Evenness) within each lineage identified by UPGMA and within each region were calculated using the “diversity_ci” function of the “Poppr” package which corrects diversity indices for sample size using rarefaction. This function was also run for linkage disequilibrium (LD) estimations for each lineage by the calculation of the index of association (Ia) and the standardized index of association (rd) with 1,000 permutations, the latter removing the dependency of Ia on the number of loci. In addition, HP-Rare 1.1. [27] was used to calculate allelic richness and private allelic richness using a rarefaction procedure.

### Analysis of gene flow pattern between T. gondii strains from Senegal and other regions of the world

Minimum spanning networks (MSN) based on Bruvo’s genetic distance were drawn using ‘‘Poppr” to visualize the relationships between *T*. *gondii* strains from Senegal and those from areas of Western Europe (France and Portugal) and Africa (Egypt, Ethiopia and South Africa) for each lineage (refer to S3 Table for genotyping data of the collection of strains used for comparative analysis). Discriminant analysis of principal components (DAPC) was used to identify genetic populations within lineage using a nonparametric approach (free from Hardy–Weinberg assumptions). In this model, genetic data were initially transformed using a principal components analysis (PCA) and subsequently clusters were identified using discriminant analysis (DA). DAPC was performed using the adegenet package [28] implemented in R version 3.4.0.

### Evaluation of strain virulence

A *T*. *gondii* strain was defined as virulent if it caused mortality in all infected mice within four weeks of bioassay or if all infected mice developed certain symptoms of acute toxoplasmosis (diminished response to handling, immobility, rapid breathing and ruffled fur) within the same period. The infecting *T*. *gondii* strain was considered as being of intermediate virulence if it caused acute infection in only a proportion of infected mice, and as non-virulent if all infected mice were asymptomatic at the end of the four weeks of monitoring. The humane endpoints of acute disease at which mice were euthanized were defined as (1) a state of fever (ruffled fur and diminished response to handling) for more than three consecutive days or (2) a state of prostration. The etiologic role of *T*. *gondii* in mortality or acute disease was confirmed by the observation of tachyzoites in peritoneal exudates punctured before euthanasia or death or in peritoneal washing made postmortem. The occurrence of a region-effect in mouse virulence was tested by Fisher’s exact test, adopting a 95% confidence interval.

## Results

Sampling followed by mouse bioassay yielded a total of 72 *T*. *gondii* isolates from the three studied regions of Senegal in infected mice (S2 Table). Microsatellite analysis of isolates revealed no mixed infection as each single isolate contained only one *T*. *gondii* strain. Sixty-eight of 72 strains could be fully genotyped at all the 15 microsatellite markers. Five additional isolates could be directly genotyped from DNA samples extracted from tissue digestate when mouse bioassay was unsuccessful. Only fully genotyped strains (n = 72) were considered in all subsequent genetic analysis.

The large majority of strains (70/72) had MLGs that were similar or identical to those of four reference strains—representing four clonal lineages—based on the allelic combination of the eight microsatellite “typing” markers that is characteristic of each lineage: ME49 (type II lineage), VEG (type III lineage), FOU (Africa 1 lineage), and TgEgCat65 (multilocus RFLP lineage ToxoDB#20). This latter lineage is described here for the first time using microsatellite markers and we designated it as “Africa 4 lineage”. Within the type II cluster, four of the 26 MLGs composing this cluster were single-repeat variants (allele 244 instead of 242 at the *W35* locus) of the ME49 type II lineage reference strain. All these four single-repeat variants of type II were confined to the city of Saint-Louis. All type III and Africa 1 MLGs from the Senegalese population strictly matched the eight “typing” alleles of the reference VEG type III and FOU Africa 1 strains, respectively. Within the Africa 4 cluster, all but one MLG (a single-repeat variant of the TgEgCat65 Africa 4 reference strain with allele 293 instead of 291 at the *TUB2* marker) shared the same alleles for the eight”typing” markers. Two MLGs (160510Gdom02 and 160517Cmos22) were composed of different admixtures of alleles compatible with a recombination between type III and Africa 1 strains at the 15 analyzed loci.

The MLG function of the “Poppr” package identified 59 MLGs among the 72 fully genotyped Senegalese strains (Table 1) and 57 of these 59 MLGs were clustered into four major groups supported by bootstrap values ≥ 60 with the UPGMA dendrogram (S1 Fig). These four clusters were largely congruent with the four groups inferred from the allelic combinations of the eight “typing” loci. In the UPGMA dendogram, the two possibly recombinant MLGs identified from the previous analysis were closer to MLGs from the type III group than those from any other group and were therefore designated as type III-*like* strains.

**Table 1:** *Toxoplasma gondii* genetic diversity and genotypic diversity. Genetic diversity and genotypic diversity were estimated for the population as a whole, per region and per lineage.

	N	MLG	eMLG	SE	A	eA	nAp	Ap	G	eG	lambda	elambda	E.5	eE.5
**Total**	72	59	9.730	0.506	6.467		-	-	49.850	6.807	0.980	0.852	0.905	0.992
**Lineages**														
type II	37	30	9.530	0.619	3.933	2.880	27	1.37	26.840	6.671	0.963	0.848	0.941	0.987
type III	7	5	5.000	0.000	1.600	1.600	5	0.38	4.450	4.455	0.776	0.776	0.931	0.931
Africa 1	13	10	7.920	0.730	2.133	1.980	3	0.46	6.760	5.168	0.852	0.793	0.769	0.908
Africa 4	13	12	9.420	0.494	2.133	1.970	13	0.94	11.270	6.611	0.911	0.847	0.961	0.984
other strain	2	2	2.000	-	-	-	1	-	-	-	-	-	-	-
**Regions**														
Dakar	23	19	14.100	0.874	4.600	4.450	13	0.94	17.060	13.005	0.941	0.922	0.941	0.953
Saint-Louis	32	27	14.800	0.871	5.067	4.640	14	0.91	24.380	13.986	0.959	0.928	0.944	0.966
Kedougou	16	12	12.000	0.000	3.533	3.533	4	0.35	8.530	8.533	0.883	0.883	0.804	0.804
Unknown	1	1	1.000	-	-	-	0	-	-	-	-	-	-	-

N, census size; MLG, multilocus genotypes; eMLG, expected MLG based on rarefaction; SE, standard error from rarefaction; A, allelic richness; eA, allelic richness based on rarefaction; nAp, number of private alleles; Ap, private allelic richness based on rarefaction; G, Stoddart and Taylor’s Index; eG, Stoddart and Taylor’s Index based on rarefaction; lambda, Simpson Index; elambda, Simpson Index based on rarefaction; E.5, Evenness; eE.5, Evenness based on rarefaction.

Type II lineage had the greatest allelic richness and private allelic richness in comparison to the three other lineages, even after applying rarefaction (n = 7) (Table 1).

Linkage disequilibrium (LD) tests revealed a strong clonal pattern by both Ia (index of association) and rd (standardized index of association) tests in all the four lineages inferred from UPGMA dendrogram (Table 2).

**Table 2:** Linkage disequilibrium of the four lineages of *Toxoplasma gondii* defined by the UPGMA dendrogram.

Lineages	Ia	p.Ia	rbarD	p.rD
type II	1.234	0.001	0.186	0.001
type III	0.516	0.043	0.129	0.048
Africa 1	0.507	0.020	0.103	0.021
Africa 4	0.538	0.011	0.138	0.011

Ia, Index of association; p.Ia, p-value for Ia; rbarD, Standardized index of association; p.rD, p-value for rbarD.

**Fig 1:**
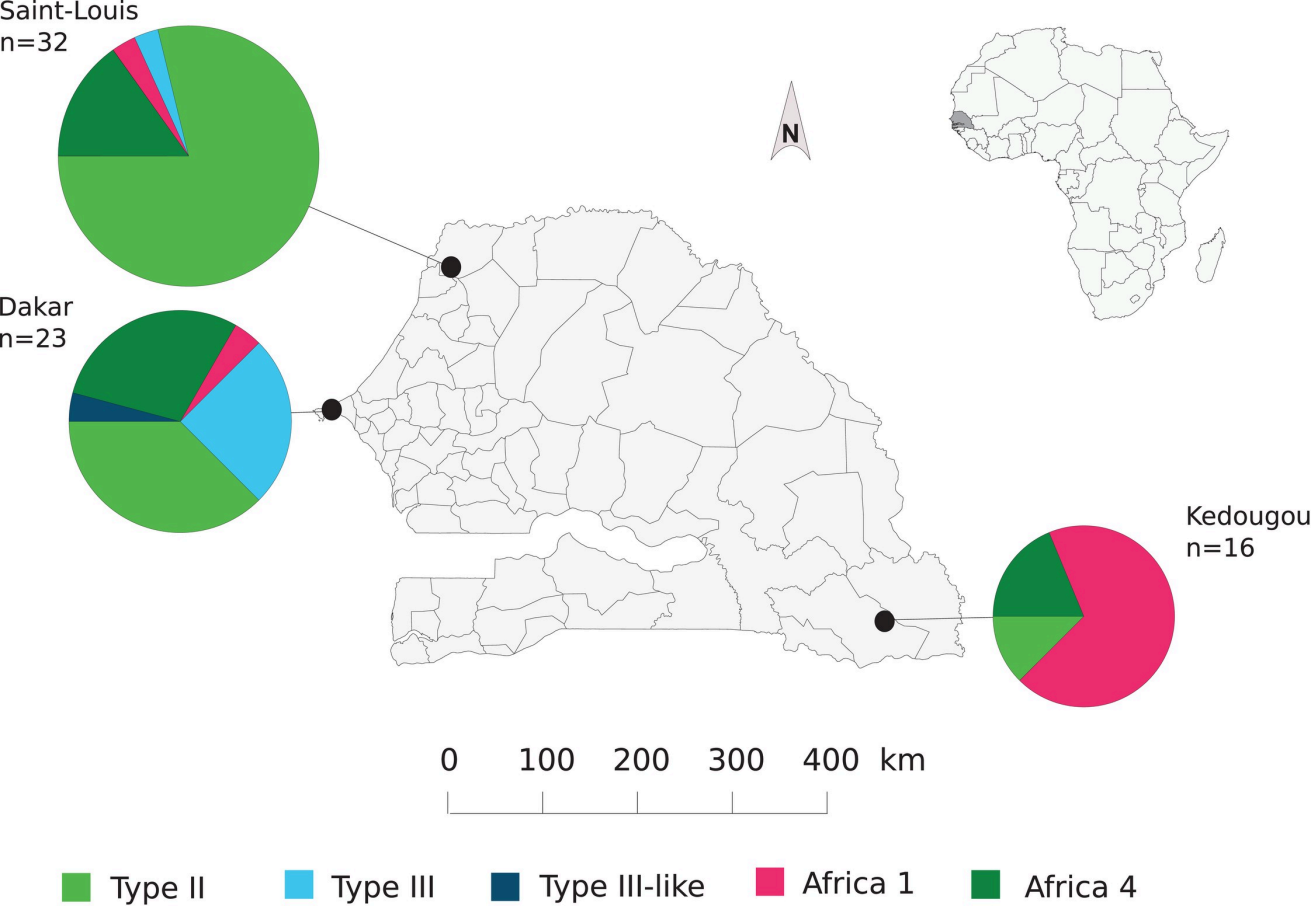
Geographical distribution of Senegalese *Toxoplasma gondii* clonal lineages and strains. Map of the distribution of *Toxoplasma gondii* clonal lineages and strains in Senegal. Black dots indicate regions from where *T*. *gondii* isolates fully genotyped were collected. Sizes of pie charts correlate with the total number of genotyped isolates (n) and colours indicate different clonal lineages of *T*. *gondii* strains.

The Africa 1 lineage was the predominant lineage in the inland region of Kedougou (Fig 1). At the opposite, type II, followed by type III and Africa 4 were the three main lineages found in both Dakar and Saint-Louis regions.

AMOVA based on geography highlighted the significant variation between regions (11.1%; p-value = 0.004), even if variation within regions accounted for most of the molecular variance (88.9%). Pairwise comparisons of regional populations showed significant differentiation between Kedougou population on the one hand and Saint-Louis (*PHI*_PT_ = 0.185; p-value = 0.006) and Dakar (*PHI*_PT_ = 0.154; p-value = 0.004) populations on the other hand, whereas Saint-Louis and Dakar populations lacked significant differentiation (*PHI*_PT_ = 0.034; p-value = 0.130). The allelic richness, private allelic richness and genotypic diversity (Simpson’s index, Stoddart and Taylor’s Index and Evenness) were greater in Dakar and Saint-Louis in comparison to Kedougou even after applying rarefaction (n = 16) (Table 1).

### Analysis of gene flow pattern between Senegal and other regions of the world

**Fig 2:**
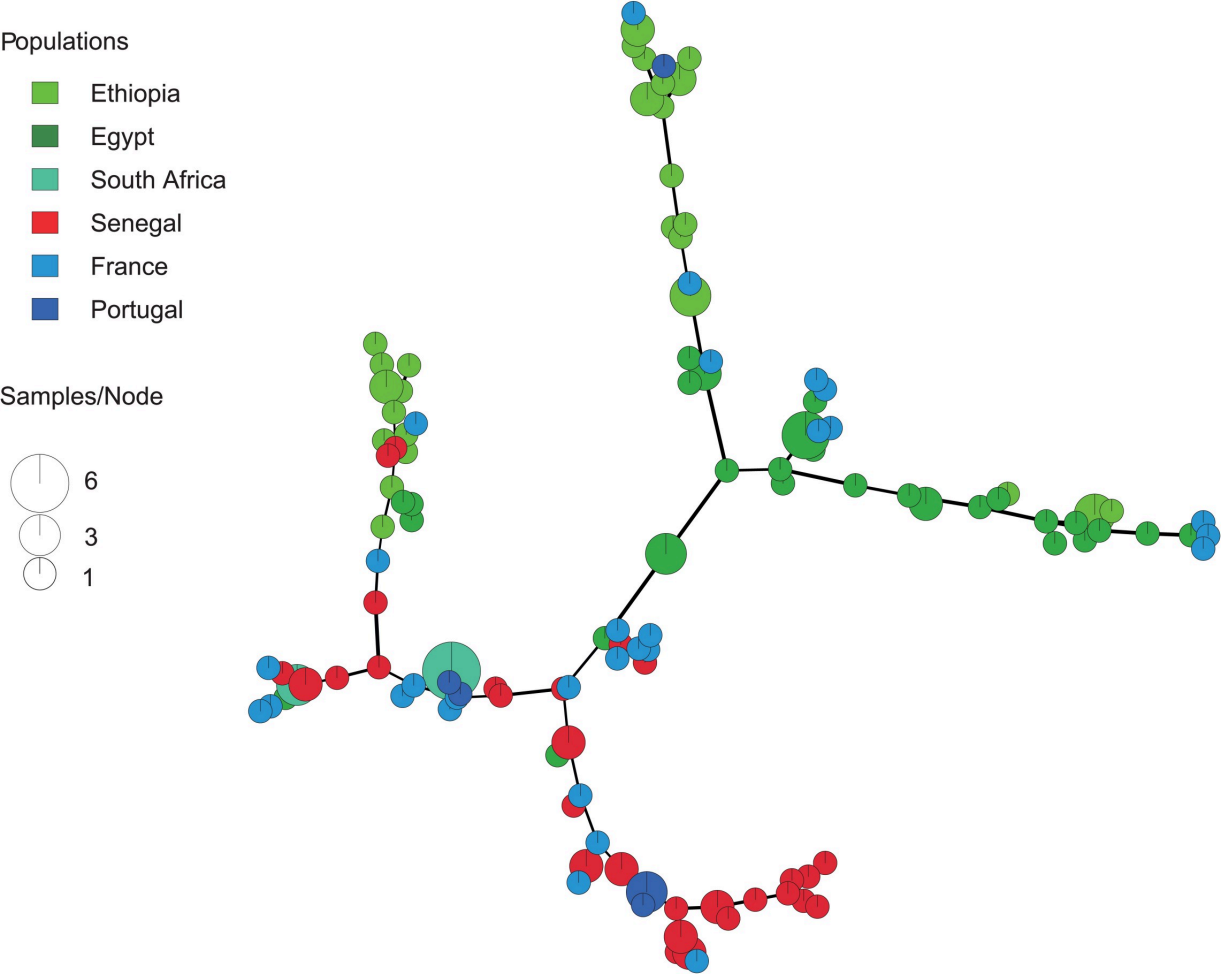
Minimum spanning network (MSN) showing the relationships between multilocus genotypes (MLGs) of type II lineage from Senegal, Western Europe, and Africa. MSNs are based on MLGs defined by 15 microsatellite markers. Each circle represents a unique MLG. The size of each circle corresponds to the number of individuals, and the colours indicate the geographical population at the country scale. Thick and dark lines show MLGs that are more closely related to each other.

The paucity of strains belonging to the type III, Africa 1 and Africa 4 lineages precluded performing extensive analysis of these lineages and hence our analyses focused on strains of type II lineage. Within the Minimum spanning networks (MSN) representing strains of type II lineage (Fig 2), Senegalese strains segregated from strains from African countries in most branches of the network and exhibited strong intermingling pattern with strains from Western Europe (France and Portugal).

**Fig 3:**
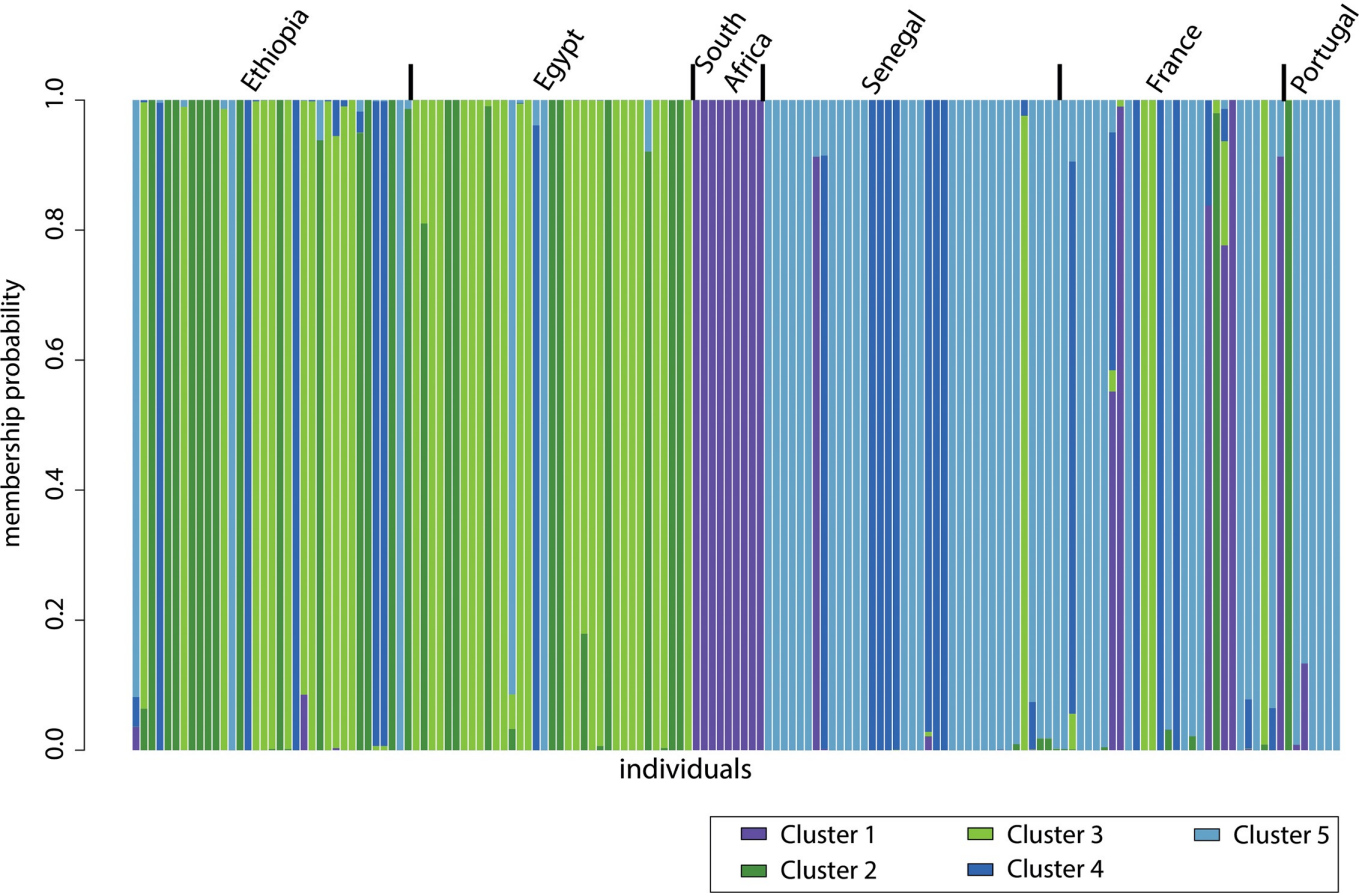
Genetic clustering of *Toxoplasma gondii* populations of type II lineage from Senegal, Western Europe, and Africa using the discriminant analysis of principal components (DAPC). Individual strains are aligned along the x-axis and grouped according to the country of origin. Strains are assigned either to one cluster (each cluster is represented by a different colour) or to multiple clusters if their genotypes were admixed (indicated by multiple colours).

Using model selection based on Bayesian information criterion (BIC) values, the optimal number of clusters was K = 5 among type II strains from Senegal, France, Portugal, Ethiopia, Egypt, and South Africa (S2 Fig). Those five clusters were differentially distributed between geographical populations (Fig 3). DAPC 1 was mainly found in South Africa. All other DAPC clusters exhibited extensive geographical distribution in Western Europe and Africa although DAPC 2 and 3 were the predominant populations in both Ethiopia and Egypt, and DAPC 5 in Senegal, France and Portugal.

### Virulence of *T*. *gondii* in mice

The virulence of *T*. *gondii* isolates in bioassayed mice varied significantly between Dakar and Saint-Louis on one hand and Kedougou on the other hand. Virulent isolates were more prevalent in Kedougou region compared to Saint-Louis (p-value < 0.001) and Dakar (p-value < 0.001) where non-virulent isolates were predominant. Lineage assignment with the eight “typing” microsatellite markers was highly predictive of virulence. All type III and Africa 4 strains, and the large majority of type II strains (34/37), caused asymptomatic infection in mice. In contrast, all mice infected by Africa 1 strains developed an acute and lethal toxoplasmosis.

## Discussion

In the present study, we found a significant differentiation between the *T*. *gondii* populations of the inland region of Kedougou and those of the port regions of Saint-Louis and Dakar. Most of *T*. *gondii* strains from Senegal could be unambiguously assigned to one of four clonal lineages: type II, type III, and Africa 1 lineages, in addition to a lineage inferred here for the first time from microsatellite analysis and designated as Africa 4 lineage. Strains belonging to this lineage were occasionally described in the literature by using RFLP markers under the genotype designation of ToxoDB#20 [29–32]. Within Senegal, although these four lineages had an extensive range of distribution and were found in all three regions (except for Kedougou where type III strains were not found), they exhibited marked regional variations in their relative abundances. In the port regions of Saint-Louis and Dakar, strains of type II lineage, followed by strains of type III and Africa 4 lineages, constituted the large majority of *T*. *gondii* strains. At the opposite, Africa 1 was by far the predominant lineage in the inland region of Kedougou. LD tests were statistically significant for all four groups, although sample sizes for each cluster were relatively low. Usually, large sample sizes are necessary to have the statistical power to reject the null hypothesis of random mating unless LD is very strong. Reaching statistical significance in LD testing for such small sample sizes indicates the robustness of the clonal structure of *T*. *gondii* populations from Senegal.

The high prevalence of strains of Africa 1 lineage in both urban and rural localities of Kedougou indicates that strains of this lineage migrate (or have migrated in the past) through terrestrial pathways between this region of Senegal and other regions of West Africa where Africa 1 is the predominant *T*. *gondii* lineage [4]. The scarce sampling of strains of Africa 1 lineage in Senegal and in other neighbouring African countries does not permit estimation of the magnitude of these migrations (rare event or extensive migrations). The introduction of this lineage in this region may have been caused by livestock transhumance, which is a millenary practice in these areas of the world. In this context, an animal that was infected in some area could be slaughtered or die hundreds of kilometers away. The carcass and offal of this animal, if consumed by local cats, could lead to the introduction of *T*. *gondii* strains into new remote areas. A possible scenario is an introduction of this lineage in Kedougou during the sedentarization of populations of Fulani nomads arriving in successive waves in this region with their livestock herds from South Mali (region of Bountou) since the end of the thirteenth century [33]. It is also possible that wildlife played a role in the regional dissemination of this lineage. In this study, an Africa 1 strain was isolated from a wild fowl of genus *Pternistis* in the region of Kedougou. It is unknown whether this wild fowl got infected from a domestic source of infection in the vicinity of human dwellings or if this lineage extensively circulates among wildlife in these areas. This latter hypothesis would be consistent with an autochthonous occurrence of this lineage in Africa. Wild strains circulating in the Amazonian rainforest of French Guiana in South America are genetically divergent from those that infect humans in populated areas bordering the forest [34] and these wild strains have often been associated with more severe disease in immunocompetent patients [6,35]. This call for further research in Africa, through collecting more strains from wildlife and characterizing the sylvatic cycle of *T*. *gondii* in this continent.

Concerning the Africa 4 lineage, its geographical pattern of distribution also suggests a terrestrial route of dissemination across an East-West axis linking Asia to Africa. Indeed, its RFLP equivalent ToxoDB#20 has been identified in China, Sri Lanka, Emirates, Egypt and Ethiopia [4,32]. In addition, the identification of strains of Africa 4 lineage in isolates of Malian and Gambian patients corroborates this scenario [4]. The caravans bringing diverse merchandise together with animals along the well-known Silk Road may have allowed the spread of this lineage between Asia and Northeast Africa [32,36,37]. From this point, dissemination in Africa through Trans-Saharan trade or livestock transhumance may explain the pattern observed in the distribution range of the Africa 4 lineage but more isolates of this lineage in Africa and especially from countries of the Sahelian belt are needed to support this hypothesis.

In Senegal, deciphering the origin of type II and type III lineages may be more challenging due to their intercontinental occurrence. In addition to a putative terrestrial propagation of these lineages following the same path as the Africa 4 lineage, an introduction from Europe through maritime trade—mediated by the invasive house mouse *Mus musculus domesticus* and the black rat *Rattus rattus*—appears to be a reasonable hypothesis given the predominance of these two lineages in Europe. Our results suggest that *T*. *gondii* type II strains from Senegal are more related to those from Western Europe than those from other areas in Africa. The port cities of Saint-Louis and Dakar are believed to be the introduction points of invasive European rodents [38–40], which may have allowed multiple transatlantic introductions of type II and type III strains in port localities. Later, the livestock chain linking inland regions to these urban poles [41] could have allowed gene flow between inland and coastal areas of the country. Since the 1930s, the development of the road infrastructure and the transport network has allowed a rapid inland dissemination of the invasive *M*. *m*. *domesticus* and *R*. *rattus*. Those species, which probably play a major role in *T*. *gondii* strains dissemination [14,16], rely on fast human means of transport like trucks for terrestrial propagation [42]. In this context, the road network development probably increased migration opportunities for *T*. *gondii* and contributed to the homogenization of *T*. *gondii* populations between the connected nodes of this network as previously shown in Gabon [21]. Kedougou region has long remained isolated from the transport network and was opened up more recently by the construction of a national road linking this area to the rest of the road network since 1998. This may have limited the exchanges between *T*. *gondii* populations of this region with those from other regions in comparison to the highly connected regions of Dakar and Saint-Louis. This assumption could explain the lower allelic and genotypic diversity found in the *T*. *gondii* populations of this region in comparison to the port regions. The occurrence of strains with lineages other than Africa 1 (Africa 4 and type II) only in urban localities of Kedougou—that are probably more exposed to exchanges through the road network than rural localities—seem to be in line with this assumption.

In port regions of Senegal, if the high prevalence of type II lineage (and to lesser extent of type III lineage) can be attributed to an introduction of these lineages through transatlantic trade, the apparently higher prevalence of Africa 4 lineage compared to Africa 1 lineage in coastal regions is more unexpected. Although the success of spread and establishment of a given lineage may be subject to random processes, it is unlikely that this mechanism solely explain the higher prevalence of Africa 4 lineage over Africa 1 lineage in both Saint-Louis and Dakar. There is experimental evidence that Africa 4, type II, and type III lineages differ markedly from Africa 1 lineage concerning mouse virulence. Africa 4, type II and type III lineages are non-virulent for laboratory mice [8,30] unless the parasite inoculum is high, whereas Africa 1 lineage leads to lethal infection in all infected mice independently from the inoculated dose of parasites [21]. In the present study, although the dose-effect could not be controlled before mouse bioassay, results of virulence in SW mice were largely congruent with results of previous studies for each of the four *T*. *gondii* lineages considered here [21,29,30,43–45]. Importantly, a recent experimental study showed that Africa 1 lineage is also lethal for wild-derived house mice *Mus musculus* [46]. Given that *M*. *m*. *domesticus* is the predominant commensal rodent in Dakar and Saint-Louis [42], we propose that this important *T*. *gondii* reservoir may favour the maintenance of non-virulent *T*. *gondii* strains in these regions, as it would die from infection by strains of the Africa 1 lineage. In line with this, results from models simulating transmission by Shwab et al. [16] support the notion that the house mouse eliminates highly virulent strains from its environment. In contrast, the native African *Mastomys natalensis* exhibits resistance to type I strains [47], which share common virulence alleles with Africa 1 strains [48]. This native African rodent, being the predominant commensal species in Kedougou [42], may consequently act as a competent reservoir for Africa 1 lineage in this region as it was previously demonstrated for other species of commensal small mammals from West Africa [49]. This may explain the contrasted geographical structure in *T*. *gondii* populations between coastal and inland regions in this study, which appear to correlate spatially with host resistance.

The most important conclusion that can be drawn from our results is that the different patterns of virulence among *T*. *gondii* strains for various reservoir hosts may be a major bottleneck for domestic *T*. *gondii* strains, driving the persistence of only certain strains in the environment, then available for human and animal infection. In the context of our study in Senegal, the human-mediated invasion of the house mouse, in addition to its putative role in the introduction of type II and type III lineages in Senegal, may be responsible of the decline of *T*. *gondii* populations of Africa 1 lineage in invaded areas. Further research should be performed to confirm the occurrence of spatial correlation between *T*. *gondii* strain virulence and murine host resistance in different geographical areas. Africa 1 lineage is one of the most prevalent lineages in West Africa, where a high prevalence of ocular toxoplasmosis has been reported among patients from this region [11,12]. The possible involvement of Africa 1 lineage in this heightened incidence of ocular toxoplasmosis has been proposed in a recent review [4]. This hypothesis is supported by the genetic proximity between Africa 1 lineage and a number of strains from South America [50,51], the continent that suffers from the highest burden of ocular toxoplasmosis [5,9,52]. By providing an accurate mapping of *T*. *gondii* lineages geographical distribution according to host species occurrence in Senegal, our findings offer a valuable framework for epidemiological studies aiming to identify the parasite determinants of ocular toxoplasmosis.

## Supporting information

S1 Table Summary of sampled animal species, bioassay trials and collected isolates. (XLSX)

S2 Table Mouse virulence evaluation and genotyping results of *Toxoplasma gondii* strains from Senegal and from eight reference strains previously collected in America, Africa, Asia, and Europe. (XLSX)

S3 Table Type II strains collected in Western Europe and Africa in previous studies. (XLSX)

S1 Fig Unweighted pair group method with arithmetic mean (UPGMA) dendrogram showing the assignment of Senegalese *Toxoplasma gondii* multilocus genotypes (MLGs) to clonal lineages. Designations of reference strains are indicated in violet italic lettering. Duplicated genotypes were removed prior to analysis and are not indicated on the distance tree. The four clonal linages identified among the Senegalese population are colour-coded (type II lineage in light green, Africa 4 lineage in dark green, Africa 1 lineage in rosy red and type III lineage in blue). Support values greater than 50% using 1,000 bootstrap samples are shown.
(EPS)

S2 Fig Genetic clustering of *Toxoplasma gondii* populations of type II lineage from Senegal, Western Europe, and Africa using the discriminant analysis of principal components (DAPC). (A) Bayesian information criterion (BIC) is provided for different numbers of clusters (from 1 to 35). (B) Scatterplot representing axes 1 and 2 of the discriminant analysis of PCA-transformed data (DAPC). Individual clones are indicated by dots. Numbers and colours represent the five genetic clusters retained from Bayesian information criterion (BIC) values.
(TIF)
