# Supplementary figures and images for: The introduction of new hosts with human trade shapes the extant distribution of Toxoplasma gondii lineages
Source: PLoS Negl Trop Dis. 2019 Jul 11;13(7):e0007435. doi: 10.1371/journal.pntd.0007435 (PMC6622481; doi:10.1371/journal.pntd.0007435)

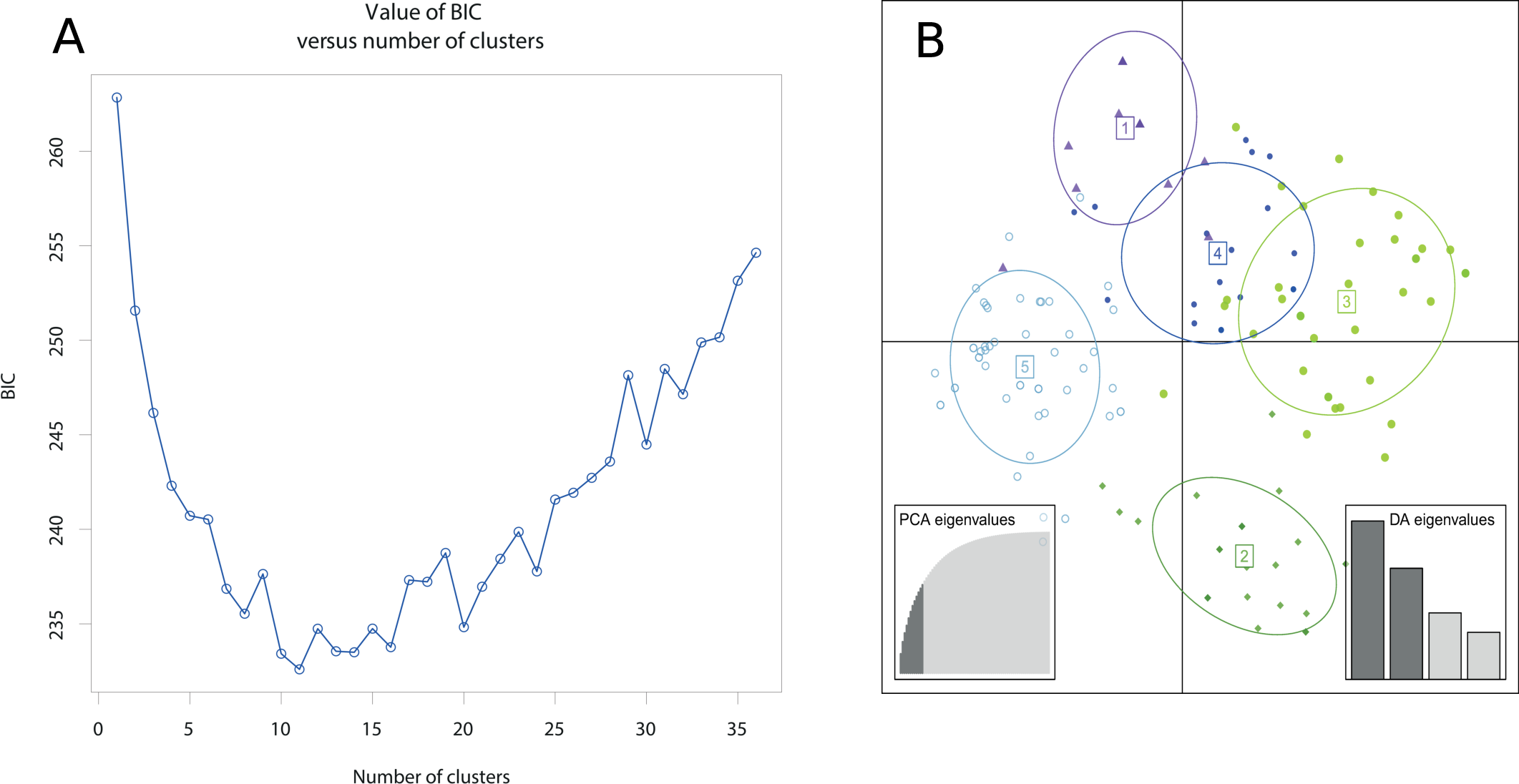

Supplement: S2 Fig — (A) Bayesian information criterion (BIC) is provided for different numbers of clusters (from 1 to 35). (B) Scatterplot representing axes 1 and 2 of the discriminant analysis of PCA-transformed data (DAPC). Individual clones are indicated by dots. Numbers and colours represent the five genetic clusters retained from Bayesian information criterion (BIC) values. (TIF) [file pntd.0007435.s005.tif]
